# Predictors of Early Posttransplant Complications and Clinical Outcomes in Kidney Transplant Recipients Aged ≥65 y

**DOI:** 10.1097/TXD.0000000000002004

**Published:** 2026-09-16

**Authors:** Daniele Vetrano, Elisa Gessaroli, Valeria Corradetti, Francesca Muccini, Marinela Skijau, Vania Cuna, Valeria Grandinetti, Chiara Abenavoli, Angelodaniele Napoletano, Claudia Bini, Chiara Zanfi, Federica Odaldi, Matteo Ravaioli, Gaetano La Manna, Giorgia Comai

**Affiliations:** 1 Department of Medical and Surgical Sciences (DIMEC), Alma Mater Studiorum-University of Bologna, Bologna, Italy.; 2 Nephrology, Dialysis and Kidney Transplant Unit, IRCCS Azienda Ospedaliero-Universitaria di Bologna, Bologna, Italy.; 3 General Surgery and Transplant Unit, IRCCS Azienda Ospedaliero-Universitaria Di Bologna, Bologna, Italy.

## Abstract

**Background.:**

Older adults represent a growing proportion of kidney transplant (KT) recipients, but early posttransplant morbidity remains substantial. We aimed to identify pretransplant factors associated with a complicated early posttransplant course in recipients aged ≥65 y and to assess its impact on subsequent outcomes.

**Methods.:**

We conducted a single-center retrospective cohort study of KT recipients aged ≥65 y. The primary in-hospital composite outcome comprised length of stay >15 d, infectious complications, delayed graft function, primary nonfunction, or in-hospital death. Patients were classified as complicated or noncomplicated. Univariable and multivariable logistic regression identified predictors of the composite outcome. Postdischarge outcomes included death, all-cause hospitalization, clinically relevant infections, surgical/urological complications, and kidney function at last follow-up. Clinical events were assessed by survival analyses and kidney function by adjusted models.

**Results.:**

Ninety-three recipients were included (median age 68.5 y; 40% aged >70 y); 61 experienced a complicated in-hospital course. In multivariable analysis, longer dialysis vintage and donor hypertension were associated with higher odds of the composite outcome, whereas preemptive transplantation and living donation were associated with lower odds. During a median follow-up of 29.8 mo, patients with a complicated early course had numerically higher rates of the composite clinical endpoint, hospitalization, and infection, but these differences were not statistically significant in Cox regression analyses. Crude estimated glomerular filtration rate was lower among patients with a complicated course; however, kidney function was comparable after adjustment for discharge estimated glomerular filtration rate.

**Conclusions.:**

Among KT recipients aged ≥65 y, longer dialysis exposure and donor hypertension were key determinants of a complicated early posttransplant course, while preemptive and living-donor transplantation appeared protective. Early complications were associated with numerically higher postdischarge event rates but were not independently associated with worse subsequent outcomes. These findings support minimizing dialysis exposure, careful donor selection, and close perioperative management in older KT recipients.

## Graphical abstract

**Figure:**
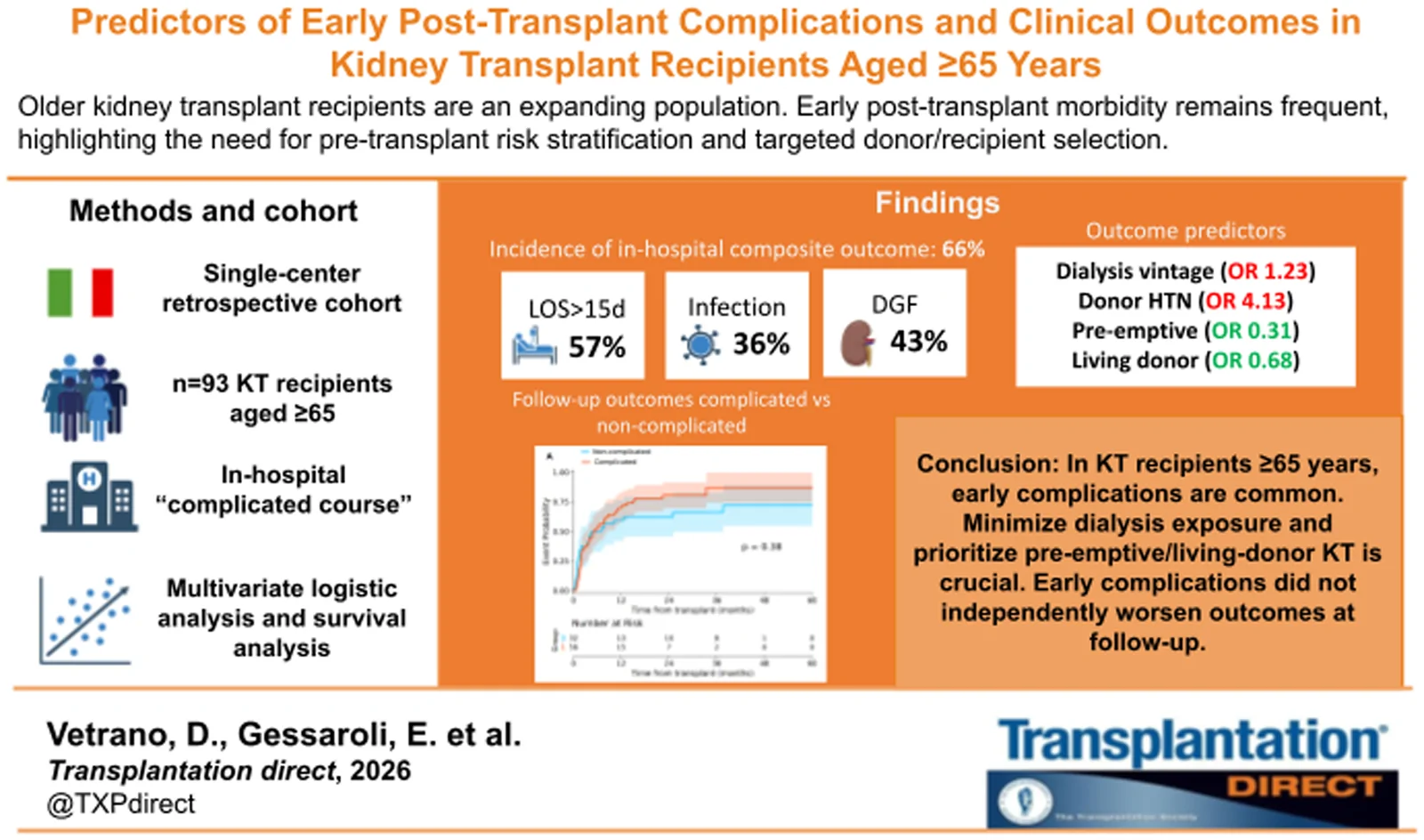

## INTRODUCTION

Chronic kidney disease (CKD) is highly prevalent and strongly age-dependent, affecting roughly 800 million adults worldwide, with an age-standardized prevalence of about 14% and rates rising steeply after the sixth decade of life.^1,2^ Population-based studies indicate that up to one-third of individuals aged ≥70 y have CKD, and that older adults account for an increasing share of those with advanced stages and on kidney replacement therapy (KRT), including dialysis and transplantation.^3,4^ Global populations are rapidly aging, with the proportion of older adults and the burden of chronic diseases steadily increasing.^5,6^ CKD is a major contributor to this trend, as its prevalence rises sharply with age and an increasing share of patients reach kidney failure in later life.^7^ Consequently, the demand for KRT in older individuals is expected to grow further in the coming decades.

Kidney transplantation (KT) is widely regarded as the optimal KRT, providing a substantial survival advantage over remaining on dialysis for waitlisted patients, with pooled estimates suggesting roughly a 50%–60% relative reduction in mortality risk.^8,9^ Beyond its clinical benefit, transplantation is also consistently the most cost-effective solution, yielding more life-years and quality-adjusted life-years at overall lower long-term costs than hemodialysis or peritoneal dialysis.^10^ Observational registry studies of patients aged ≥65–70 y show that KT offers a clear long-term survival benefit compared with remaining on dialysis. In matched analyses, older transplant recipients reach 5- and 10-y survival rates of roughly 75%–80% and 50%–55%, versus about 50%–55% and 15%–20% in waitlisted patients who stay on dialysis, corresponding to an overall 40%–60% reduction in mortality risk.^11,12^ However, the early posttransplant period remains clinically vulnerable in older recipients, particularly among those receiving extended-criteria donor kidneys, in whom an increased short-term mortality risk has been reported.^13^

These observations highlight the importance of identifying, among older candidates, those at increased risk of a complicated posttransplant course. Emerging data suggest that comorbidity burden, cardiovascular disease, functional status, frailty, and donor or organ quality may all modulate outcomes in elderly recipients, but pretransplant risk stratification in this age group remains suboptimal.^14,15^

On this basis, we conducted a retrospective study aimed at identifying pretransplant clinical and functional characteristics associated with a “complicated” posttransplant course in recipients aged ≥65 y and to evaluate whether such an early complicated course was associated with postdischarge clinical outcomes and graft function during the available follow-up.

## MATERIALS AND METHODS

### Study Design and Population

We designed a single-center, retrospective observational cohort study conducted at the Nephrology, Dialysis, and Transplantation Unit of Sant’Orsola University Hospital in Bologna, Italy. We included all consecutive KTs performed between January 1, 2021, and May 31, 2024, in recipients aged ≥65 y with at least 12 mo of posttransplant follow-up (or until death, if earlier). All transplant procedures were performed by the same dedicated transplant surgical team, with longstanding experience at our center, according to standardized surgical techniques and postoperative surgical care pathways. Both single and dual KTs, as well as deceased- and living-donor grafts, were eligible. The study adhered to the Declaration of Helsinki and was approved by the local Ethics Committee (protocol 586/2023/Oss/AOUBo). All patients provided written informed consent.

To identify pretransplant recipient and donor factors associated with an in-hospital complicated course, we defined a composite endpoint as the occurrence of at least one of the following events during early posttransplant hospitalization: prolonged length of stay (>15 d), documented infectious complication, delayed graft function (DGF) or primary nonfunction (PNF), or in-hospital death. Prolonged length of stay was defined as >15 d, corresponding approximately to the mean length of stay plus 1 SD in our larger historical adult KT cohort, including nonelderly recipients, indicating a clinically complicated course. Although no formalized protocol or prespecified discharge checklist was in place during the study period, a consistent center-specific clinical practice was applied across the cohort: patients were generally discharged only after graft function had stabilized or was clearly improving and after active infections, surgical or urological complications, and other conditions requiring inpatient monitoring or treatment had been adequately resolved. Based on this composite outcome, patients were classified as having a “complicated” (primary outcome present) or “noncomplicated” (primary outcome absent) early posttransplant course.

Then, using this in-hospital classification (complicated versus noncomplicated), we compared posttransplant clinical outcomes between the 2 groups throughout the available follow-up. The composite follow-up outcome included all-cause mortality, all-cause hospitalizations, clinically relevant infections (defined as bacterial or viral infections requiring specific antimicrobial or antiviral treatment), and major surgical or urological complications. In exploratory analyses, we also evaluated the frequency of all-cause mortality, biopsy-proven acute rejection, graft failure, and death-censored graft failure (DCGF). Follow-up events were assessed from the date of discharge after the index transplant hospitalization. Events occurring during the initial hospitalization were not reclassified as follow-up events; in particular, patients were not routinely discharged with active unresolved infections or major surgical/urological complications, and postdischarge events therefore represented newly incident complications requiring clinical evaluation, treatment, hospitalization, or procedural management. Lastly, we evaluated graft function at the last available follow-up, expressed as estimated glomerular filtration rate (eGFR), comparing patients with and without an in-hospital complicated course.

### Data Collection and Variables

Data were obtained from the institutional electronic medical records. For each eligible recipient, we retrospectively collected pretransplant demographic and clinical variables, including age, sex, ethnicity, primary kidney disease, dialysis vintage and modality, waitlist time, comorbidities, cardiovascular history, type of KRT, body mass index (BMI), and immunological profile, including any previous transplant and maximum calculated panel reactive antibody. Laboratory parameters closest to transplantation were also recorded. Donor and transplant-related variables included donor age, sex, BMI, hypertension and diabetes, donor type (living, donation after brain death [DBD] or donation after circulatory death [DCD]), extended-criteria donor status, single versus dual kidney allocation, cold ischemia time, induction regimen, and maintenance immunosuppression. For deceased-donor grafts only, the Kidney Donor Profile Index and Kidney Donor Risk Index values were also collected.

Perioperative and in-hospital data included early graft function, expressed as serum creatinine, eGFR, and 24-h proteinuria at discharge; additionally, we recorded the occurrence of DGF or PNF, respectively, defined as the requirement for at least 1 dialysis session within the first 7 d posttransplant (excluding dialysis performed for nongraft-related postoperative reasons such as volume overload and hyperkalemia) and permanent nonfunction of a transplanted kidney within the first 90 d of transplant.^9,16^ Moreover, we collected any documented infectious, surgical, or urological complications, and length of hospital stay. Follow-up data, including survival status, all-cause hospitalizations, clinically relevant infections, major surgical or urological complications, and graft function expressed as serum creatinine and eGFR, were retrieved from discharge summaries, outpatient visit notes, and microbiology or imaging reports.

### Statistical Analysis

Baseline recipient, donor, and transplant characteristics were summarized overall and by in-hospital primary outcome status (complicated versus noncomplicated). Continuous variables were expressed as mean ± SD or median and interquartile range, as appropriate, and compared using Student *t* test or Wilcoxon rank-sum test. Categorical variables were presented as counts and percentages and compared using chi-square or Fisher exact test.

For the primary analysis, we explored factors associated with the in-hospital composite outcome using univariable and multivariable logistic regression. Candidate predictors included recipient-related variables and donor/transplant characteristics. Univariable odds ratios (ORs) with 95% confidence intervals (CIs) were first estimated for each covariate. Variables with *P* < 0.10 in univariable analyses, or considered clinically relevant, were included in a multivariable model. A reduced final model was then obtained using backward stepwise selection, taking care to limit overfitting. Results are reported as adjusted ORs with 95% CIs. Because the individual components of the in-hospital composite outcome may reflect partly distinct clinical pathways, we additionally performed exploratory component-specific analyses for prolonged hospitalization, documented infectious complications, and DGF. PNF and in-hospital death were not analyzed separately because of the very low number of events.

For posttransplant clinical outcomes, time-to-event analyses were performed for the composite endpoint and each of its components, comparing patients with and without an in-hospital complicated course. Time was calculated from the date of transplantation to the first occurrence of the outcome of interest or censoring at the last available follow-up. Kaplan-Meier curves were generated and compared using the log-rank test, and hazard ratios (HRs) with 95% CIs were estimated using Cox proportional hazards models. Given the limited number of events, death, biopsy-proven acute rejection, graft loss, and DCGF were summarized descriptively and were not included in comparative time-to-event models when event numbers or distribution across groups did not allow reliable estimation.

In addition, to compare longer-term kidney function between groups, we performed an ANCOVA, modeling eGFR at the last available follow-up as the dependent variable, in-hospital outcome status (complicated versus noncomplicated) as the main factor, and eGFR at discharge as a covariate. Adjusted mean eGFR at the last available follow-up with 95% CIs, β coefficients, F statistics, and *P*-values were derived from this model.

All tests were 2-sided, and a *P*-value of <0.05 was considered statistically significant. Analyses were conducted using R (Version 4.5.2; R Foundation for Statistical Computing, Vienna, Austria).

## RESULTS

### Baseline Characteristics

Baseline recipient, donor, and transplant characteristics according to the in-hospital primary outcome are shown in Table 1.

**TABLE 1.** Baseline recipient, donor, and transplant characteristics according to in-hospital composite outcome (noncomplicated versus complicated course)

	Noncomplicated (N = 32)	Complicated (N = 61)	Total (N = 93)	*P*
Age, y, median (IQR)	68.00 (65.75–72.00)	69.00 (67.00–71.00)	68.50 (66.00–72.00)	0.5
Age >70, n (%)	12 (37.5)	25 (41.7)	37 (40.2)	0.6
Sex, n (%)				0.7
Male	24 (75.0)	44 (72.1)	68 (73.1)	
Female	8 (25.0)	17 (27.9)	25 (26.9)	
Caucasian ethnicity, n (%)	32 (100.0)	60 (98.4)	92 (98.9)	0.4
BMI, kg/m^2^, mean (±SD)	24.35 (3.99)	25.61 (2.92)	25.17 (3.36)	0.08
BMI class, n (%)				
18.5–25 kg/m^2^	17 (53.1)	25 (40.9)	42 (45.2)	0.3
>25 kg/m^2^	15 (46.9)	36 (59.1)	51 (54.8)	0.2
Hypertension, n (%)	30 (93.8)	59 (96.7)	89 (95.7)	0.5
Diabetes, n (%)	5 (15.6)	14 (23.0)	19 (20.4)	0.4
CCI, mean (±SD)	4.97 (1.96)	5.23 (1.90)	5.14 (1.91)	0.5
Kidney disease, n (%)				0.6
Diabetic nephropathy	2 (6.2)	2 (3.3)	4 (4.3)	
Glomerulonephritis	12 (37.5)	19 (31.1)	31 (33.3)	
Genetic	5 (15.6)	8 (13.1)	13 (14.0)	
Unknown	6 (18.8)	20 (32.8)	26 (28.0)	
Other	7 (21.9)	12 (19.7)	19 (20.4)	
Dialysis time, mo, median (IQR)	32.23 (10.22–45.27)	41.80 (25.88–63.80)	40.23 (21.83–59.43)	0.033
Waitlist time, mo, median (IQR)	8.73 (5.05–23.65)	23.03 (5.65–39.66)	16.50 (5.20–34.20)	0.06
Dialysis type, n (%)				0.06
PD	8 (29.6)	8 (13.3)	16 (18.4)	
HD	19 (70.4)	52 (86.7)	71 (81.6)	
Preemptive, n (%)	5 (15.6)	1 (1.6)	6 (6.5)	0.009
Living donor, n (%)	11 (34.4)	7 (11.5)	18 (19.4)	0.008
DCD, n (%)	0 (0.0)	11 (20.0)	11 (14.5)	0.02
Dual transplant, n (%)	15 (46.9)	35 (57.4)	50 (53.8)	0.3
Second transplant, n (%)	2 (6.2)	4 (6.8)	6 (6.6)	0.9
Maximum cPRA, median (IQR)	0.00 (0.00–5.00)	1.50 (0.00–31.25)	0.00 (0.00–10.25)	0.2
Donor age, y, median (IQR)	71.00 (66.00–76.00)	74.00 (66.00–78.00)	73.00 (66.00–77.00)	0.9
Donor sex, n (%)				0.4
Male	16 (50.0)	25 (41.7)	41 (44.6)	
Female	16 (50.0)	35 (58.3)	51 (55.4)	
Donor BMI, kg/m^2^, mean (±SD)	25.72 (4.40)	26.09 (4.98)	25.98 (4.78)	0.7
Donor hypertension, n (%)	12 (48.0)	39 (70.9)	51 (63.8)	0.048
Donor diabetes, n (%)	2 (8.0)	7 (12.5)	9 (11.1)	0.5
Extended criteria donor, n (%)	14 (70.0)	38 (76.0)	52 (74.3)	0.6
KDPI, median (IQR)	82.00 (69.25–95.00)	94.00 (78.00–97.00)	92.00 (75.00–96.50)	0.4
KDRI, median (IQR)	1.36 (1.18–1.70)	1.63 (1.30–1.79)	1.59 (1.27–1.76)	0.2
Cold ischemia time, h, median (IQR)	10.33 (3.52–12.41)	11.83 (9.00–14.58)	11.38 (8.54–13.25)	0.019
Induction regimen, n (%)				0.086
Basiliximab	16 (53.3)	27 (45.8)	43 (48.3)	
ATG	14 (46.7)	32 (54.2)	46 (51.7)	
Time to discharge, d, median (IQR)	12.00 (10.50–13.00)	21.00 (16.00–31.00)	16.00 (13.00–27.00)	<0.001
sCreatinine, mg/dL, median (IQR)	1.25 (1.02–1.71)	1.94 (1.41–2.90)	1.69 (1.27–2.48)	0.06
eGFR, mL/min/1.73 m^2^, median (IQR)	61.00 (34.50–68.50)	30.50 (19.00–42.00)	38.00 (23.00–56.25)	<0.001
Proteinuria, mg/24 h, median (IQR)	720.00 (585.75–1179.50)	520.00 (324.00–950.00)	676.00 (402.50–970.00)	0.1
Maintenance IS, n (%)				0.09
FK-MPA-steroid	22 (71.0)	42 (72.4)	64 (71.9)	
Steroid free	8 (25.8)	5 (8.6)	13 (14.6)	
Other	1 (3.2)	11 (18.0)	12 (12.9)	

ATG, anti-thymocyte thymoglobulin; BMI, body mass index; CCI, Charlson Comorbidity Index; cPRAs, calculated panel reactive antibodies; DCD, donation after circulatory death; eGFR, estimated glomerular filtration rate; FK, tacrolimus; HD, hemodialysis; IQR, interquartile range; IS, immunosuppression; KDPI, Kidney Donor Profile Index; KDRI, Kidney Donor Risk Index; MPA, mycophenolic acid; PD, peritoneal dialysis; sCreatinine, serum creatinine.

**FIGURE 1.**
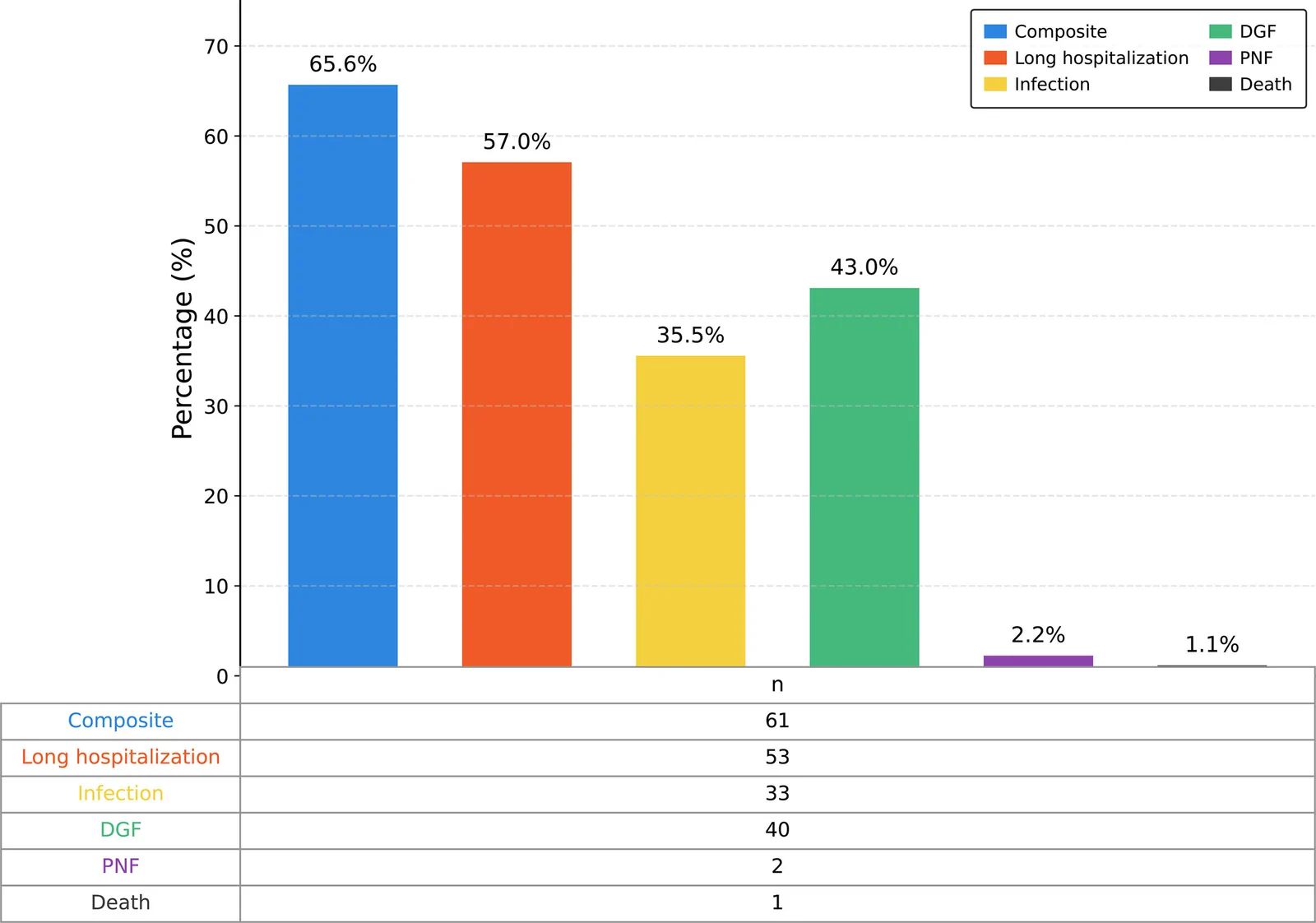
Incidence of the in-hospital composite outcome and its individual components in the overall cohort. DGF, delayed graft function; PNF, primary nonfunction.

Overall, 93 KT recipients aged ≥65 y were included, with a median age of 68.5 y, 40% of patients were >70 y, 73% were male, and almost all were of Caucasian ethnicity. Overall, 61 of 93 recipients (65.6%) met the in-hospital composite outcome (complicated group; Figure 1). The most frequent component was prolonged hospitalization (>15 d), which occurred in 53 patients (57.0%), followed by DGF in 40 (43.0%) and documented infections in 33 (35.5%). PNF and in-hospital death were rare, being observed in 2 (2.2%) and 1 (1.1%) patient, respectively (Figure 1).

Comorbidities were similar between groups, but dialysis vintage tended to be longer in recipients in the complicated group (*P* = 0.033). Preemptive transplantation and living donation were more frequent among noncomplicated patients (preemptive: 16% versus 2%; *P* = 0.009 and living donor: 34% versus 12%; *P* = 0.008), whereas DCD was observed only in the complicated group (0% versus 20%; *P* = 0.02). Median pretransplant dialysis time was 40.2 mo overall and was longer among recipients who experienced a complicated in-hospital course than among those without complications (41.8 versus 32.2 mo; *P* = 0.033).

Donor age was high in both groups (median ~73 y) and did not differ significantly, as did donor sex, BMI, diabetes, and vascular disease. However, donor hypertension was more common among recipients with a complicated course (71% versus 48%; *P* = 0.048). Most grafts met extended criteria and had high Kidney Donor Profile Index/Kidney Donor Risk Index scores, with no relevant between-group differences. Cold ischemia time was slightly longer in recipients with the in-hospital primary outcome (*P* = 0.019).

Induction and initial maintenance immunosuppression regimens were broadly similar between the 2 groups.

At hospital discharge, patients with the in-hospital primary outcome tended to have lower graft function, with lower median eGFR (30.5 versus 61.0 mL/min/1.73 m^2^; *P* < 0.001), while 24-h proteinuria did not differ significantly (Table 1).

### Risk Factors for In-hospital Primary Outcome

In univariable logistic regression (Table 2), longer dialysis vintage (OR, 1.26; 95% CI, 1.02-1.60) and longer waitlist time (OR, 1.28; 95% CI, 1.01-1.74) were associated with a higher risk of outcome. Preemptive transplantation (OR, 0.09; 95% CI, 0.01-0.59) and living donation (OR, 0.24; 95% CI, 0.08-0.71) were associated with substantially lower odds, whereas DCD donation (versus DBD; OR, 2.65; 95% CI, 1.15-4.56), donor hypertension (OR, 2.64; 95% CI, 1.01-7.13; *P* = 0.04), and longer cold ischemia time (OR, 1.14; 95% CI, 1.03-1.27) were associated with higher odds of an in-hospital primary outcome.

**TABLE 2.** Univariable and multivariable logistic regression analyses of factors associated with the in-hospital composite outcome

Characteristic	Univariate		Multivariate	
	OR (95% CI)	*P*	OR (95% CI)	*P*
Age, y	1.03 (0.90-1.19)	0.5	1.24 (1.00-1.60)	0.06
Age >70, yes/no	1.19 (0.49-2.92)	0.6		
Sex, male/female	0.86 (0.31-2.24)	0.7	0.89 (0.20-2.84)	0.7
BMI, kg/m^2^	1.12 (0.98-1.30)	0.07		
BMI >25 kg/m^2^, yes/no	1.63 (0.69-3.90)	0.2		
HTN, yes/no	1.96 (0.22-17.05)	0.5		
Diabetes, yes/no	1.60 (0.54-5.41)	0.4		
CCI	1.07 (0.85-1.38)	0.5		
Preemptive, yes/no	0.09 (0.01-0.59)	0.03^*a*^	0.31 (0.21-0.68)	0.02^*a*^
Dialysis time, mo	1.26 (1.02-1.60)	0.03^*a*^	1.23 (1.01-1.78)	0.048^*a*^
Dialysis type, PD/HD	0.36 (0.11-1.12)	0.07		
Waitlist time, mo	1.28 (1.01-1.74)	0.048^*a*^		
Donor type, living/deceased	0.24 (0.08-0.71)	0.01^a^	0.68 (0.44-0.89)	0.03^*a*^
Donor type, DCD/DBD	2.65 (1.15-4.56)	0.04^*a*^		
Donor type, double/single	1.52 (0.64-3.64)	0.3		
Donor age, y	1.00 (0.96-1.03)	0.9	0.98 (0.85-1.03)	0.3
Donor HTN, yes/no	2.64 (1.01-7.13)	0.04^*a*^	4.13 (1.08-17.31)	0.041^*a*^
Donor diabetes, yes/no	1.64 (0.36-11.60)	0.5		
KDPI score	1.00 (0.98-1.02)	0.4		
KDRI score	2.46 (0.62-10.17)	0.2		
Cold ischemia time, h	1.14 (1.03-1.27)	0.01^*a*^	1.39 (0.36-24.8)	0.9
Induction therapy, ATG/basiliximab	1.35 (0.56-3.30)	0.4		

ATG, anti-thymocyte thymoglobulin; BMI, body mass index; CCI, Charlson Comorbidity Index; CI, confidence interval; DBD, donation after brain death; DCD, donation after circulatory death; HD, hemodialysis; HTN, hypertension; KDPI, Kidney Donor Profile Index; KDRI, Kidney Donor Risk Index; OR, odds ratio; PD, peritoneal dialysis.

a Indicates statistical significance (*P* < 0.05).

**FIGURE 2.**
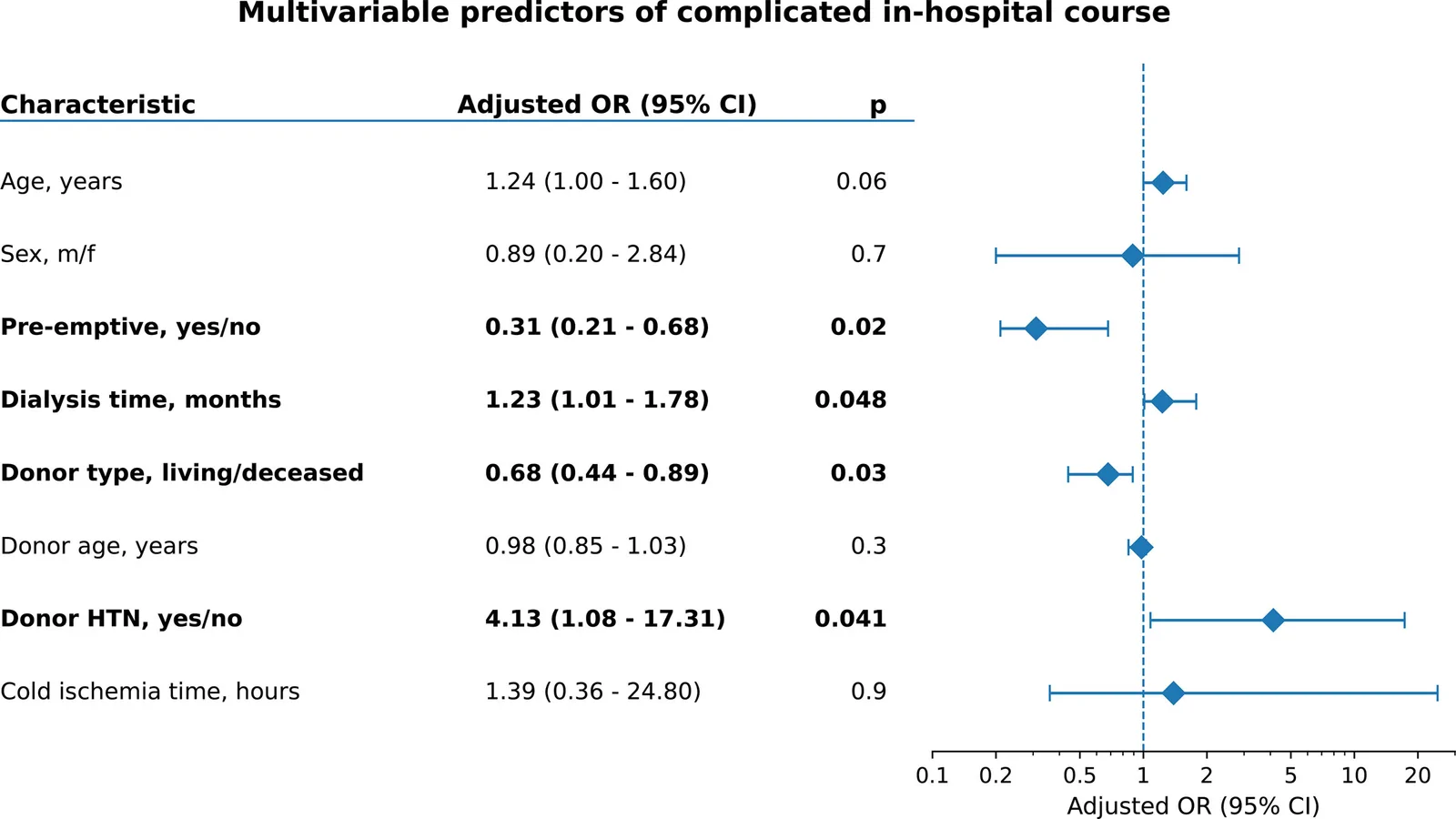
Multivariable predictors of a complicated early in-hospital course in kidney transplant recipients aged ≥65 y. CI, confidence interval; f, female; HTN, hypertension; m, male; OR, odds ratio.

In the multivariable model (Table 2; Figure 2), preemptive transplantation (OR, 0.31; 95% CI, 0.21-0.68) and living donation (OR, 0.68; 95% CI, 0.44-0.89) remained independently associated with lower odds of a complicated early course. Longer dialysis vintage was associated with a higher risk (OR per year, 1.23; 95% CI, 1.01-1.78), as was donor hypertension (OR, 4.13; 95% CI, 1.08-17.31). There was also a borderline association between higher recipient age and increased risk, whereas cold ischemia time was no longer significant after adjustment. Analyses of the individual components of the composite outcome are reported in **Table S1** (**SDC**, https://links.lww.com/TXD/A901). Although several associations overlapped across outcomes, the pattern of predictors differed according to the component considered, supporting the interpretation that prolonged hospitalization, infectious complications, and DGF represent related but clinically distinct manifestations of early posttransplant morbidity.

### Clinical Outcomes and Graft Function During Follow-up

**FIGURE 3.**
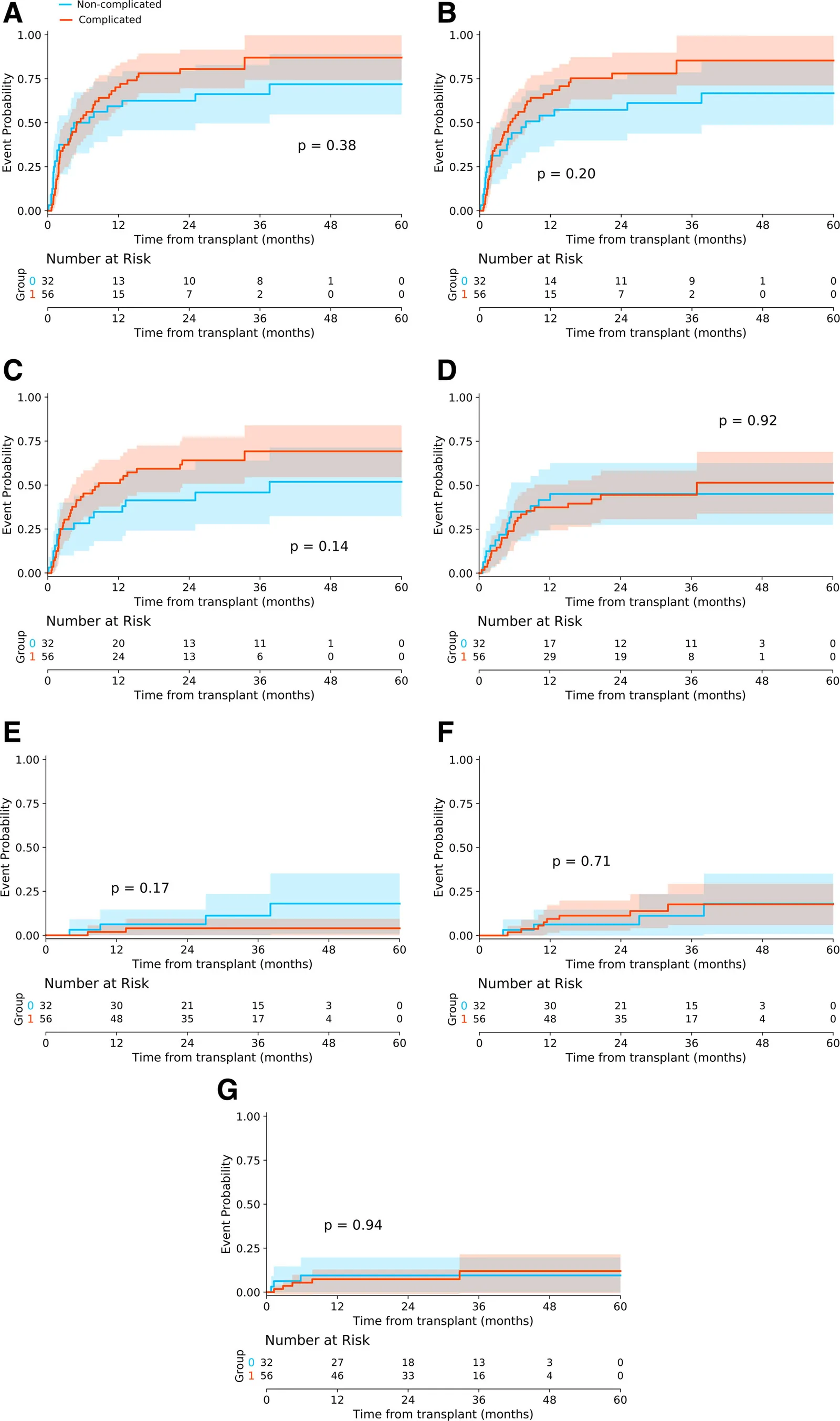
Kaplan-Meier curves for postdischarge outcomes according to in-hospital course. A, Composite outcome. B, All-cause hospitalization. C, Infectious complications. D, Surgical/urological complications. E, Death. F, Graft loss. G, Biopsy-proven acute rejection.

During a median follow-up of 29.8 mo (interquartile range, 21.4–40.3 mo; maximum, 58.3 mo), the composite follow-up outcome occurred in 22 of 32 recipients (68.8%) in the noncomplicated group and in 44 of 56 recipients (78.6%) in the complicated group. All-cause hospitalization occurred in 20 of 32 (62.5%) and 42 of 56 (75.0%) recipients, infectious events in 15 of 32 (46.9%) and 35 of 56 (62.5%), and surgical/urological complications in 14 of 32 (43.8%) and 24 of 55 (43.6%), respectively. Despite the numerically higher crude event frequencies in the complicated group for the composite outcome, hospitalizations, and infections, Kaplan-Meier curves and Cox proportional hazards models did not show statistically significant between-group differences: (HR, 1.26; 95% CI, 0.75-2.11; *P* = 0.39) for the composite outcome, (HR, 1.41; 95% CI, 0.82-2.43; *P* = 0.21) for all-cause hospitalization, (HR, 1.57; 95% CI, 0.85-2.89; *P* = 0.15) for infectious events, and (HR, 0.97; 95% CI, 0.50-1.87; *P* = 0.92) for surgical/urological complications (Figure 3; **Table S2** [**SDC**, https://links.lww.com/TXD/A901]). Sensitivity analyses excluding living-donor recipients, DCD recipients, and both groups combined (therefore restricted to DBD recipients) yielded results consistent with the main analysis, with no statistically significant differences in postdischarge composite outcomes, hospitalizations, infections, or surgical/urological complications according to the in-hospital course (**Table S2**, **SDC**, https://links.lww.com/TXD/A901).

During follow-up, death occurred in 4 of 32 recipients (12.5%) in the noncomplicated group and in 2 of 56 recipients (3.6%) in the complicated group. In contrast, biopsy-proven acute rejection occurred in 3 of 32 (9.4%) and 5 of 56 (8.9%) recipients, respectively. Graft loss occurred in 4 of 32 recipients (12.5%) in the noncomplicated group and in 8 of 56 (14.3%) in the complicated group. DCGF occurred in 6 recipients (10.7%), all in the complicated group. However, given the low number of events, comparative Cox modeling was not considered reliable.

**FIGURE 4.**
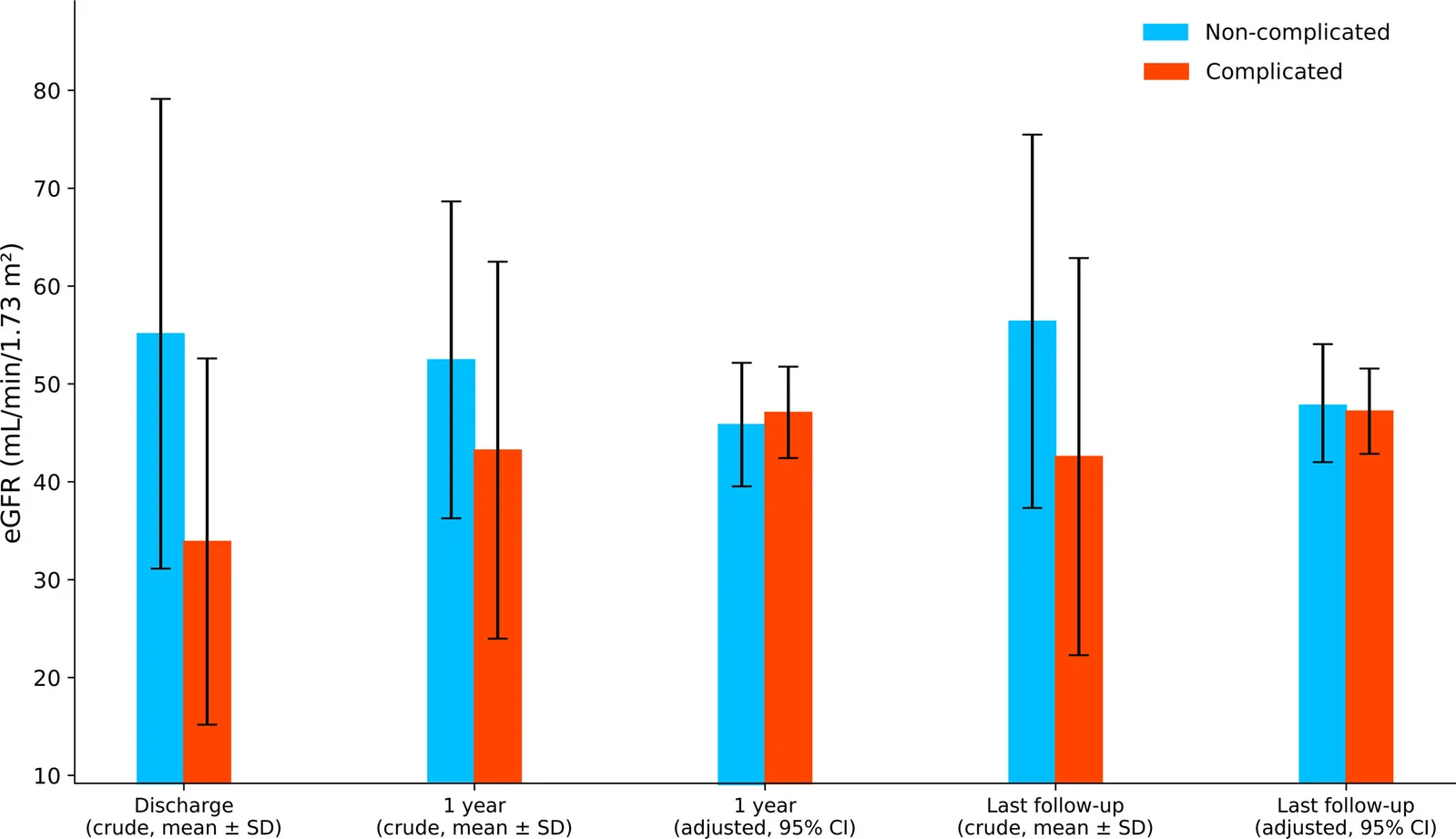
Kidney function according to in-hospital course. Crude eGFR is shown at discharge, 1 y, and last available follow-up. Adjusted eGFR estimates are shown at 1 y and last available follow-up after adjustment for discharge eGFR. Error bars represent SD for crude values and 95% CI for adjusted estimates. CI, confidence interval; eGFR, estimated glomerular filtration rate.

At the last available follow-up, crude mean eGFR was higher in patients without an in-hospital complicated course (56.4 versus 42.6 mL/min/1.73 m^2^; *P* = 0.003). However, after adjustment for discharge eGFR (Figure 4; **Table S3** [**SDC**, https://links.lww.com/TXD/A901]), mean eGFR was similar between groups (48.0 versus 47.2 mL/min/1.73 m^2^; adjusted difference, –0.8 mL/min/1.73 m^2^; 95% CI, –8.6 to 7.0; *P* = 0.835), while discharge eGFR remained strongly associated with eGFR at the last available follow-up (β = 0.62 per 1 mL/min/1.73 m^2^; 95% CI, 0.46-0.79; *P* < 0.001).

## DISCUSSION

This single-center study of KT recipients aged ≥65 y shows that early posttransplant complications are frequent, with almost two-thirds of patients meeting our in-hospital composite outcome. We identified several pretransplant recipient and donor factors associated with a higher risk of a complicated early course, including longer dialysis vintage and donor hypertension, whereas preemptive and living-donor transplantation were associated with lower risk. Despite a higher incidence of adverse events at follow-up in patients with an early complicated course, neither survival analyses nor graft function showed clear prognostic separation between groups.

Although the survival and quality-of-life benefits of KT over remaining on dialysis are well established even in recipients aged ≥65–70 y, older adults are consistently reported to have a more complex early posttransplant course, with higher rates of perioperative complications and infections.^11,12^ However, long-term outcomes appear broadly comparable to those of younger transplant recipients, supporting the offering of transplantation to older candidates despite the early procedural risk. The observation that nearly two-thirds of recipients aged ≥65 y experienced an in-hospital complicated course is consistent with prior evidence and reinforces the importance of more precise risk stratification to identify older candidates most vulnerable to early posttransplant morbidity. Dialysis vintage emerged as a significant predictor of early complications, whereas neither individual comorbidities nor overall comorbidity burden, as measured by the Charlson Comorbidity Index, showed a similar association. This finding indicates that dialysis vintage should be regarded as an important contributor to patient frailty and perioperative vulnerability, particularly in elderly candidates. Registry studies have associated longer dialysis vintage with worse posttransplant outcomes, with higher rates of early complications and increased long-term mortality compared with preemptive or short-vintage transplantation.^17-19^ Indeed, its role deserves careful consideration during the pretransplant assessment, where standardized frailty scoring is not yet routinely applied. To further support this observation, our results also suggest that preemptive transplantation is associated with better in-hospital outcomes in older recipients. This pattern is consistent with at least 1 other study in elderly recipients, in which preemptive transplantation was likewise associated with fewer early complications and better graft outcomes.^20^

Among donor-related factors, receiving a kidney from a living donor was independently associated with a lower risk of an in-hospital complicated course in our cohort, in line with existing literature.^21^ This likely reflects the combined effect of shorter dialysis and waitlist exposure, better organ quality, and shorter cold ischemia times typically observed with living donation. Moreover, our observation is consistent with large registry analyses in elderly recipients, which similarly report superior graft survival and better-preserved renal function after living-donor transplantation compared with both DBD and DCD.^22^ Conversely, in our cohort, DCD donation was associated with the in-hospital composite outcome in univariable analyses, a relationship that is likely mediated by the higher incidence of DGF and longer ischemia times typically observed with DCD grafts.^23^

In our cohort, most donor features were not independently associated with the in-hospital composite outcome. By contrast, a history of donor hypertension was associated with higher odds of early complications. This finding is in line with previous cohort studies showing donor hypertension to be an independent risk factor for both early complications and long-term graft outcomes.^24-27^ Chronic hypertensive injury is associated with nephrosclerosis, reduced functional nephron mass, and increased susceptibility to ischemia-reperfusion injury, which may render kidneys from hypertensive donors particularly vulnerable when transplanted in older recipients.^28,29^

At follow-up, patients with an in-hospital complicated course showed a higher incidence of the composite endpoint and of each of its components, but these differences did not reach statistical significance in survival analyses. This pattern is likely influenced by the limited sample size and number of events, which reduces power to detect moderate risk differences. However, it is also consistent with previous reports in older transplant recipients, where the main excess risk is concentrated in the early posttransplant period, while medium-term outcomes tend to become similar between higher- and lower-risk patients.^30^

Moreover, we did not observe significant differences in adjusted follow-up eGFR. The higher crude eGFR at follow-up in the noncomplicated group mainly reflected the lower eGFR at discharge in the complicated group, which notably improved during follow-up. Furthermore, no significant difference in rejection events was detected between the 2 groups, probably reflecting the strict and proactive follow-up strategy required to balance the comorbidity burden with the persistent risk of rejection, which remains relevant in older transplant recipients.^31-33^

From a clinical perspective, these findings should not be interpreted as grounds to deny transplantation to older candidates. Rather, they support individualized candidate selection and adequate perioperative management. Recent evidence in transplant candidates aged ≥75 y similarly suggests that deceased-donor KT carries an early mortality risk but may provide a survival benefit after an initial break-even period of approximately 3 y, underscoring the need to balance early perioperative risk against potential longer-term benefit.^34^ This balance is particularly relevant because chronological age alone may not adequately capture biological reserve, frailty, or vulnerability to early complications. In this context, careful donor evaluation, individualized donor-recipient matching, and strategies aimed at minimizing dialysis exposure, such as preemptive or living-donor transplantation, may help optimize outcomes in this population. Our findings also support the incorporation of standardized frailty assessment into the pretransplant evaluation to better identify those at increased risk of complications and to guide tailored perioperative and postdischarge care, while also helping to identify potentially modifiable vulnerabilities before transplantation.

In this regard, recent randomized evidence suggests that structured exercise-based prehabilitation can improve exercise capacity, muscle strength, and muscle size in KT candidates, including frail patients.^35^ Although our study did not include standardized frailty measures or prehabilitation interventions, these data support the potential value of integrating functional assessment and targeted pretransplant optimization strategies into the evaluation of KT candidates. Notably, in our cohort, an early complicated in-hospital course was not associated with significantly worse postdischarge clinical outcomes or adjusted graft function during extended follow-up, suggesting that complications, when promptly recognized and appropriately managed, may not necessarily translate into poorer longer-term outcomes. Larger prospective studies are needed to validate and implement more comprehensive pretransplant risk-assessment tools in older KT candidates.

## LIMITATIONS AND STRENGTHS

Several limitations should be acknowledged. First, the retrospective single-center design and relatively small sample size, together with the low number of hard clinical events, limited statistical precision and the generalizability of our findings. This limitation was further compounded by heterogeneity in donor and transplant characteristics, including living, DBD, and DCD donation, as well as single and dual KT. Accordingly, nonsignificant findings should not be interpreted as evidence of the absence of clinically meaningful associations. Although follow-up was extended to the last available clinical assessment and yielded results consistent with the initial analyses, longer observation does not fully compensate for the limited sample size and event number.

Second, residual confounding cannot be excluded. Relevant characteristics of older transplant candidates, including frailty, performance status, cognitive impairment, and social support, were not systematically available. In addition, although all procedures were performed by the same dedicated transplant surgical team using shared surgical and postoperative care pathways, detailed intraoperative variables, including operative duration, hemodynamic instability, anesthetic management, and unforeseen surgical complications, were not captured with sufficient completeness for analysis. These unmeasured factors may have influenced DGF, infection, and length of stay.

Third, the in-hospital composite endpoint was intentionally designed as a pragmatic measure of early posttransplant morbidity. DGF was included because it reflects clinically relevant perioperative graft injury and, when prolonged, has been associated with acute rejection and DCGF after transplantation.^36^ However, the individual components may reflect partly distinct clinical pathways. In particular, prolonged hospitalization may be influenced by center-specific discharge practices and organizational factors; nevertheless, such practices may vary across transplant programs.

Finally, the high proportion of dual KT reflects local recipient-selection and donor-allocation practices. Candidacy for dual transplantation is restricted to recipients aged >55 y, while kidneys from older deceased donors may be preferentially allocated as dual rather than single grafts according to donor characteristics and expected nephron mass. In addition, age-matching allocation preferentially directs kidneys from older donors toward older recipients. These features may limit the applicability of our findings to centers using different allocation strategies.

Despite these limitations, this study has several strengths. It focuses on a contemporary cohort of KT recipients aged ≥65 y, including a substantial proportion of recipients aged >70 y, and combines detailed recipient, donor, and transplant information. By linking a pragmatic in-hospital morbidity classification with postdischarge clinical outcomes and graft function during extended follow-up, it provides a clinically relevant picture of how pretransplant and perioperative characteristics may shape early posttransplant morbidity in older adults. The analysis of graft function adjusted for discharge eGFR further helps clarify that an initially complicated in-hospital course did not necessarily translate into poorer subsequent graft function.

## CONCLUSIONS

In recipients aged 65 y or older, KT can achieve favorable outcomes during follow-up. Although individuals with an initially complicated in-hospital course experienced more subsequent adverse events, postdischarge clinical outcomes and adjusted graft function remained comparable to those of recipients with an uncomplicated course, suggesting that timely recognition and effective management of early complications may mitigate their longer-term impact. Careful donor evaluation, including appropriate selection of hypertensive or DCD grafts, together with strategies aimed at minimizing dialysis exposure such as preemptive or living-donor transplantation, may be particularly important in this population. Larger prospective studies should validate comprehensive pretransplant risk-assessment tools, including standardized frailty measures, to better identify vulnerable candidates and guide individualized transplant management, from donor-recipient matching to perioperative care.

## Supplementary Material


