# Supplementary material for: Predictors of Early Posttransplant Complications and Clinical Outcomes in Kidney Transplant Recipients Aged ≥65 y
Source: Transplant Direct. 2026 Sep 16;12(10):e2004. doi: 10.1097/TXD.0000000000002004 (PMC13593112; doi:10.1097/TXD.0000000000002004)
Supplement: Supplementary file 1 [file txd-12-e2004-s001.pdf]

Supplementary Table S1. Univariable logistic regression analyses for secondary in-hospital outcomes (long hospitalization, infectious complications, and delayed graft function).

	Long Hospitalization			Infectious complications			DGF		
**Characteristic**	**OR**	**95% CI**	**p-value**	**OR**	**95% CI**	**p-value**	**OR**	**95% CI**	**p-value**
Age, *years*	0.95	0.84-1.09	0.5	0.95	0.82-1.08	0.4	1.06	0.93-1.21	0.3
Age>70, *yes/no*	0.70	0.30-1.63	0.4	0.87	0.35-2.10	0.7	1.37	0.59-3.22	0.4
Sex, *m/f*	1.05	0.41-2.66	0.9	1.63	0.61-4.68	0.3	0.97	0.38-2.48	0.9
BMI, *Kg/m2*	1.00	0.88-1.13	0.9	1.05	0.92-1.19	0.4	1.09	0.96-1.25	0.1
BMI>25 kg/m2, y*es/no*	0.98	0.43-2.25	0.9	0.94	0.40-2.24	0.8	1.04	0.45-2.40	0.9
BMI<20 kg/m2, *yes/no*	0.22	0.03-1.02	0.07	0.23	0.01-1.39	0.1	0.16	0.01-0.98	0.009*
Liver disease, *yes/no*	6.93	1.19-131.53	0.07	4.14	1.01-20.85	0.04*	0.62	0.12-2.52	0.5
HTN, *yes/no*	1.34	0.15-11.59	0.7	0.54	0.06-4.71	0.5	1.56	0.14-34.28	0.7
Diabetes, *yes/no*	1.84	0.65-5.72	0.2	2.77	1.01-8.16	0.04	1.05	0.36-2.96	0.9
Prior tumor, *yes/no*	0.54	0.10-2.59	0.4	2.57	0.53-13.81	0.2	0.97	0.18-4.67	0.9
CCI	1.04	0.84-1.31	0.6	1.29	1.03-1.67	0.03	0.98	0.77-1.22	0.8
Pre-emptive, *yes/no*	0.13	0.01-0.88	0.007*	0.33	0.01-2.21	0.3	0.75	0.55-0.98	0.03*
Dialysis time, *years*	1.19	1.01-1.47	0.04*	1.37	1.13-1.70	0.002*	1.22	1.03-1.49	0.02*
Dialysis type, *PD/HD*	0.32	0.10-0.98	0.01*	0.19	0.02-0.75	0.03	0.46	0.13-1.42	0.1
Waitlist time, *years*	1.09	0.88-1.39	0.4	1.24	1.02-1.64	0.03	1.10	0.88-1.37	0.3
Calcimimetic use, *yes/n0*	5.06	0.82-98.26	0.1	2.66	0.55-14.32	0.2	3.50	0.70-25.42	0.1
Donor type, *living/decease*	0.33	0.10-0.98	0.04*	0.29	0.06-0.98	0.04*	0.34	0.12-0.55	0.01*
Donor type, *DCD/DBD*	7.56	1.33-14.48	0.03*	0.28	0.04-1.21	0.1	2.75	0.72-13.41	0.1
Donor type, *double/single*	1.30	0.57-2.99	0.5	0.50	0.20-1.19	0.1	2.61	1.12-6.31	0.02
Donor age, *years*	1.00	0.96-1.04	0.7	0.95	0.91-0.99	0.03*	1.03	0.99-1.08	0.07
Donor HTN, *yes/no*	3.4	1.32-9.04	0.01*	1.26	0.49-3.35	0.6	4.28	1.63-12.12	0.004*
Donor diabetes, *yes/no*	1.42	0.34-7.19	0.6	1.30	0.30-5.34	0.7	0.77	0.17-3.17	0.7
KDPI score	1.01	0.99-1.03	0.2	0.98	0.96-1.01	0.09	1.02	1.01-1.05	0.04*
KDRI score	2.33	0.65-8,80	0.1	0.32	0.08-1.14	0.08	3.50	0.99-13.70	0.058
Ischemia time, *hours*	1.11	1.01-1.22	0.02*	1.12	1.02-1.25	0.01*	1.14	1.04-1.28	0.006*
Induction therapy, *thymo/basi*	1.12	0.48-2.61	0.6	0.93	0.38-2.22	0.8	1.62	0.69-3.84	0.2

Supplementary Table S2. Main and Sensitivity Survival Analyses of Post-Discharge Outcomes According to In-Hospital Course

Analysis	Outcome	Non-complicated, n/N (%)	Complicated, n/N (%)	HR (95% CI)	Cox p-value	Log-rank p-value
**Main analysis**<br>Median follow-up: 29.8 months (IQR 21.4–40.3)	Composite outcome	22/32 (68.8)	44/56 (78.6)	1.26 (0.75–2.12)	0.387	0.385
	All-cause hospitalization	20/32 (62.5)	42/56 (75.0)	1.41 (0.82–2.43)	0.210	0.207
	Infectious events	15/32 (46.9)	35/56 (62.5)	1.57 (0.85–2.89)	0.148	0.145
	Surgical/urological complications	14/32 (43.8)	24/55 (43.6)	0.97 (0.50–1.87)	0.924	0.924
**Excluding living-donor recipients**<br>Median follow-up: 29.1 months (IQR 19.2–38.4)	Composite outcome	14/21 (66.7)	38/50 (76.0)	1.42 (0.76–2.65)	0.265	0.262
	All-cause hospitalization	13/21 (61.9)	36/50 (72.0)	1.48 (0.78–2.81)	0.235	0.232
	Infectious events	10/21 (47.6)	30/50 (60.0)	1.48 (0.72–3.04)	0.290	0.286
	Surgical/urological complications	8/21 (38.1)	19/49 (38.8)	0.99 (0.43–2.25)	0.972	0.972
**Excluding DCD recipients**<br>Median follow-up: 29.7 months (IQR 20.8–41.1)	Composite outcome	22/32 (68.8)	38/45 (84.4)	1.42 (0.83–2.42)	0.198	0.195
	All-cause hospitalization	20/32 (62.5)	36/45 (80.0)	1.57 (0.90–2.75)	0.109	0.106
	Infectious events	15/32 (46.9)	31/45 (68.9)	1.82 (0.98–3.39)	0.058	0.055
	Surgical/urological complications	14/32 (43.8)	23/45 (51.1)	1.18 (0.61–2.30)	0.619	0.619
**DBD recipients only (excluding living-donor and DCD recipients)**<br>Median follow-up: 28.4 months (IQR 18.7–39.0)	Composite outcome	14/21 (66.7)	32/39 (82.1)	1.63 (0.86–3.08)	0.134	0.130
	All-cause hospitalization	13/21 (61.9)	30/39 (76.9)	1.65 (0.86–3.20)	0.135	0.130
	Infectious events	10/21 (47.6)	26/39 (66.7)	1.73 (0.83–3.61)	0.142	0.137

	Surgical/urological complications	8/21 (38.1)	18/39 (46.2)	1.23 (0.53–2.82)	0.633	0.632

Supplementary Table S3. ANCOVA of eGFR at 12 months and at the last available follow-up according to in-hospital course, adjusted for discharge eGFR.

Outcome and model term	Adjusted mean eGFR (95% CI)	Difference (95% CI)	β coefficient (95% CI)	F	p-value
**eGFR at 12 months**					
Non-complicated	45.9 (39.5 to 52.2)	Ref.	Ref.		
Complicated	47.1 (42.4 to 51.8)	+1.2 (−7.0 to +9.5)	+1.24 (−7.04 to +9.52)	0.089	0.766
Covariate: discharge eGFR	—	—	+0.48 (+0.30 to +0.66)	29.4	<0.001
**eGFR at last available follow-up**					
Non-complicated	48.0 (42.0 to 54.1)	Ref.	Ref.		
Complicated	47.2 (42.9 to 51.6)	−0.8 (−8.6 to +7.0)	−0.82 (−8.63 to +6.98)	0.044	0.835
Covariate: discharge eGFR	—	—	+0.62 (+0.46 to +0.79)	55.2	<0.001
